# Neutrophil Extracellular Traps in Breast Cancer: Roles in Metastasis and Beyond

**DOI:** 10.7150/jca.94669

**Published:** 2024-04-23

**Authors:** Xi Xu, Xinyu Wang, Zhuomeng Zheng, Yuxuan Guo, Guangchun He, Yian Wang, Shujun Fu, Chanjuan Zheng, Xiyun Deng

**Affiliations:** Key Laboratory of Translational Cancer Stem Cell Research, Department of Pathophysiology, Hunan Normal University School of Medicine, Changsha, Hunan, China.

**Keywords:** Neutrophil extracellular trap (NET), Breast cancer, Tumor immunity, Therapeutic target, Tumor microenvironment

## Abstract

Despite advances in the treatment of breast cancer, the disease continues to exhibit high global morbidity and mortality. The importance of neutrophils in cancer development has been increasingly recognized. Neutrophil extracellular traps (NETs) are web-like structures released into the extracellular space by activated neutrophils, serving as a potential antimicrobial mechanism for capturing and eliminating microorganisms. The roles played by NETs in cancer development have been a subject of intense research in the last decade. In breast cancer, current evidence suggests that NETs are involved in various stages of cancer development, particularly during metastasis. In this review, we try to provide an updated overview of the roles played by NETs in breast cancer metastasis. These include: 1) facilitating systemic dissemination of cancer cells; 2) promoting cancer-associated inflammation; 3) facilitating cancer-associated thrombosis; 4) facilitating pre-metastatic niche formation; and 5) awakening dormant cancer cells. The translational implications of NETs in breast cancer treatment are also discussed. Understanding the relationship between NETs and breast cancer metastasis is expected to provide important insights for developing new therapeutic strategies for breast cancer patients.

## 1. Introduction

Breast cancer has become the most common malignancy worldwide, with the highest incidence and mortality rates among women globally, and metastasis being the primary cause of most breast cancer-related deaths 1. At each stage of this complex process, malignant cells must also confront and resist attacks from the host immune system. Despite advances in targeted therapy and immunotherapy, breast cancer, particularly triple-negative breast cancer (TNBC), still exhibits high invasiveness, metastasis, and recurrence 2. Therefore, the search for novel therapeutic strategies in breast cancer is of paramount importance.

Neutrophils are the most abundant white blood cells in the bloodstream, comprising up to 70% of circulating white blood cells in humans 3, 4. They serve as the frontline warriors against invading microorganisms and are a major component of the body's innate immune system 5. Tumor-associated neutrophils (TANs) have been increasingly recognized as significant contributors to tumor biology. Neutrophils paradoxically play a tumor-suppressive or tumor-promoting role in cancer immunobiology, depending on the states of neutrophils and the specific context involved 6. The importance of neutrophils in diverse types of cancer including breast cancer is further illustrated by a recent study indicating that neutrophils exhibit diverse cancer-related functions including inflammation, angiogenesis, and antigen presentation 7.

In 2004, Brinkmann *et al.* observed a unique form of neutrophil degranulation, termed neutrophil extracellular trap (NET), as a novel mechanism for neutrophils to fight against microorganisms 8. In 2012, Demers *et al.* first reported the role of NETs in cancer showing that hematological as well as solid tumors induce an increase in peripheral blood neutrophils that are associated with NET formation 9. Since then, it has been widely accepted that NETs play a crucial role in the development of various types of malignancies 10. The roles of NETs in breast cancer have been extensively studied in the last decade, which have become a subject of several nice reviews 5, 11-13. In this review, we summarize recent advances regarding the roles of NETs in breast cancer with a special focus on metastasis-related events.

## 2. Pathways of NET Formation

**Figure 1:**
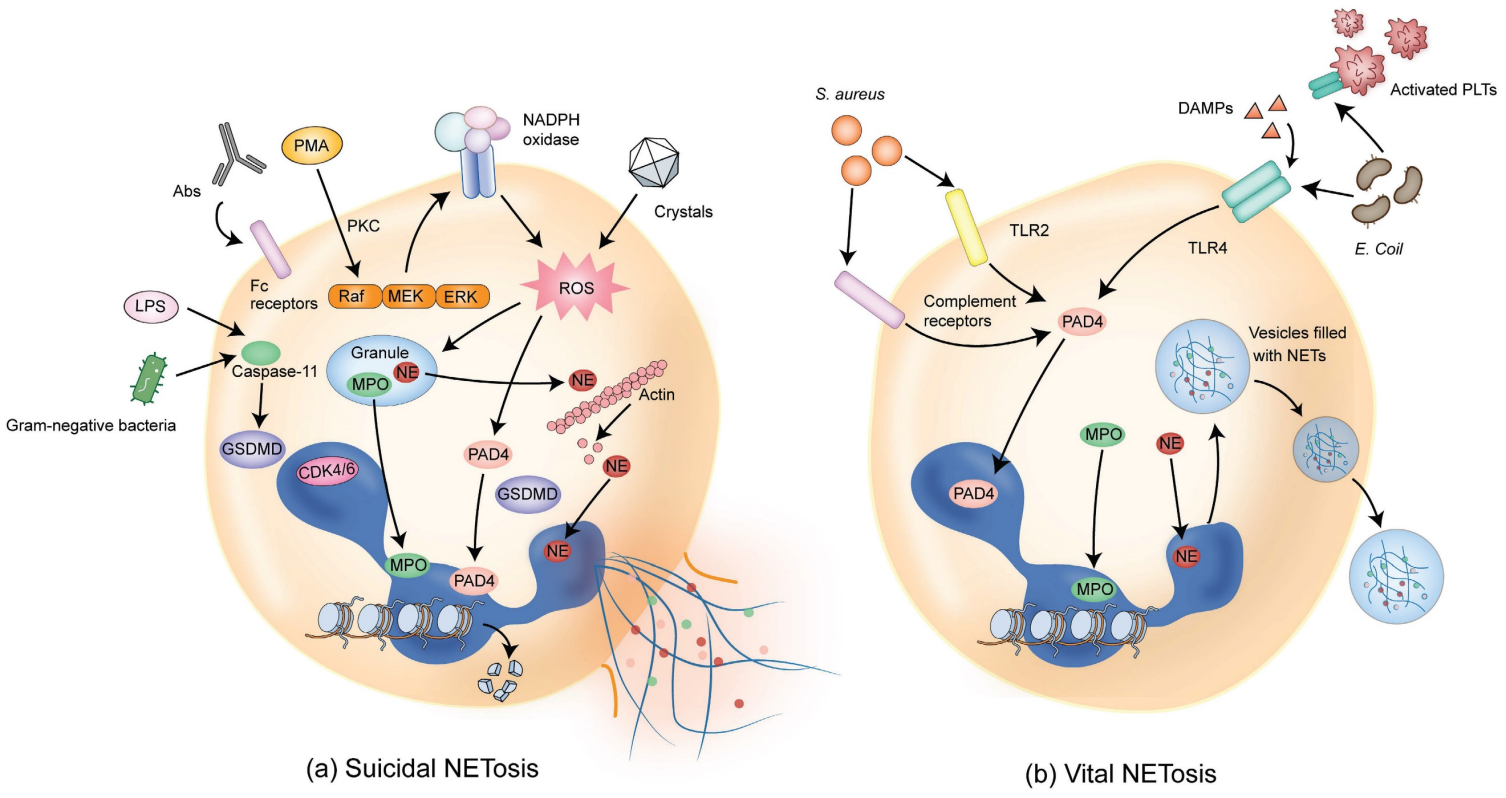
** Pathways of NET formation.** (a) Different stimuli such as PMA, antibodies (such as autoantibodies), or cholesterol crystals can induce suicidal NETosis, which occurs a few hours after stimulation. Following the activation of NOX, ROS are generated and PAD4 is activated, followed by chromatin decondensation. Subsequently, NE and MPO are translocated from granules to the nucleus, promoting further chromatin unfolding and subsequent disruption of the nuclear membrane. In addition, NE activates GSDMD, forming pores on granules and plasma membranes, resulting in membrane rupture and cell death. (b) Vital NETosis can be induced by *Staphylococcus aureus* within minutes through complement receptors and TLR2 ligands. Alternatively, vital NETosis can be induced via TLR4 activation by *Escherichia coli*. PLTs indirectly activated through TLR4 also contribute to this process. In this pathway, NOX activity is not required. PAD4 is activated, inducing chromatin decondensation. Nuclei containing DNA are extruded without disrupting the plasma membrane, and decondensed chromatin is transported via vesicles for the expulsion of nuclear DNA. The intact cell membrane of neutrophils allows for the survival of non-nucleated neutrophils while retaining physiological functions such as phagocytosis. DAMPs that amplify ongoing immune reactions are also involved in inducing vital NETosis. PMA, phorbol 12-myristate 13-acetate; NOX, NADPH oxidase; ROS, reactive oxygen species; PAD4, peptidylarginine deiminase 4; NE, neutrophil elastase; MPO, myeloperoxidase; GSDMD, Gasdermin D; TLR2, Toll-like receptor 2; PLTs, platelets; TLR4, Toll-like receptor 4; DAMPs, damage-associated molecular patterns.

NETs are composed of DNA, histones, and granule proteins such as neutrophil elastase (NE), myeloperoxidase (MPO), cathepsin G, and other enzymatically active proteins. Current evidence suggests that two primary pathways are involved in the formation of NETs. The first pathway, termed suicidal or lytic NETosis (**Figure 1a**), occurs when stimulated by triggers such as phorbol 12-myristate 13-acetate (PMA) and extracellular microbes 6, 14-16. This pathway involves the stimulation of protein kinase C (PKC) and Raf-MEK-ERK signaling, activating NADPH oxidase (NOX) and the production of reactive oxygen species (ROS) in neutrophils 17-19. Upon ROS stimulation, NE escapes from the granules and translocates to the nucleus, where NE cleaves histones and promotes chromatin decondensation. Subsequently, MPO binds to chromatin and collaborates with NE to induce plasma membrane rupture and NET formation 20.

Furthermore, the activation of peptidylarginine deiminase 4 (PAD4), possibly together with the cell cycle proteins such as CDK4/6, promotes histone cleavage and chromatin decondensation through citrullination 21-23 and initiates nuclear envelope rupture and subsequently membrane rupture 24, 25. Cytoplasmic NE also activates Gasdermin D (GSDMD), which is known to be able to form pores in the granule membrane and the plasma membrane during pyroptosis 26, promoting NE release into the cytoplasm and assisting in NET expulsion 27. With the rupture of the cell membrane, the chromatin decorated with granule proteins is released into the extracellular space, forming NETs.

The second type of NETosis, termed vital or non-lytic NETosis (**Figure 1b**), involves the expulsion of nuclear chromatin accompanied by the release of granule proteins through degranulation. This type of cell death-independent NETosis occurs more rapidly, typically within minutes, and can be stimulated by triggers such as activated platelets, microorganisms including *Staphylococcus aureus*, and complement proteins 28-30. In this pathway, NOX activity is not required and DNA-containing nuclear vesicles are extruded without disrupting the plasma membrane, thus preserving neutrophil functionality 6, 31.

While both pathways result in the formation of web-like structures that trap microorganisms or non-infectious pathogens, several differences exist in NET formation between suicidal and vital NETosis 32. First, the nature of the stimulation is different between these two pathways. Suicidal NETosis is mostly induced by PMA, whereas vital NETosis is stimulated by microbial-specific molecular patterns and recognized by host pattern recognition receptors, such as toll-like receptors (TLRs). Second, different signaling pathways are involved in the two pathways. Suicidal NETosis requires activation of the Raf-MEK-ERK pathway and NOX-dependent production of ROS. In contrast, vital NETosis is ROS-independent and requires vesicular trafficking of DNA from within the nucleus to the extracellular space. Third, plasma membrane rupture is required and occurs in suicidal but not vital NETosis. Fourth, there exists difference in the time duration required for the formation of NETs. Vital NETosis executes more rapidly compared with suicidal NETosis (< 1 hour vs 2 - 4 hours). Fifth, the outcomes are different between suicidal and vital NETosis. Neutrophils undergoing suicidal NETosis could no longer be recruited and, therefore, cannot be used for sustainable host immune defense. On the contrary, vital NETosis sustains neutrophil functionality related to innate immunity in terms of detecting, capturing, and restraining target microorganisms 32.

## 3. Roles of NETs in Breast Cancer Metastasis

**Figure 2:**
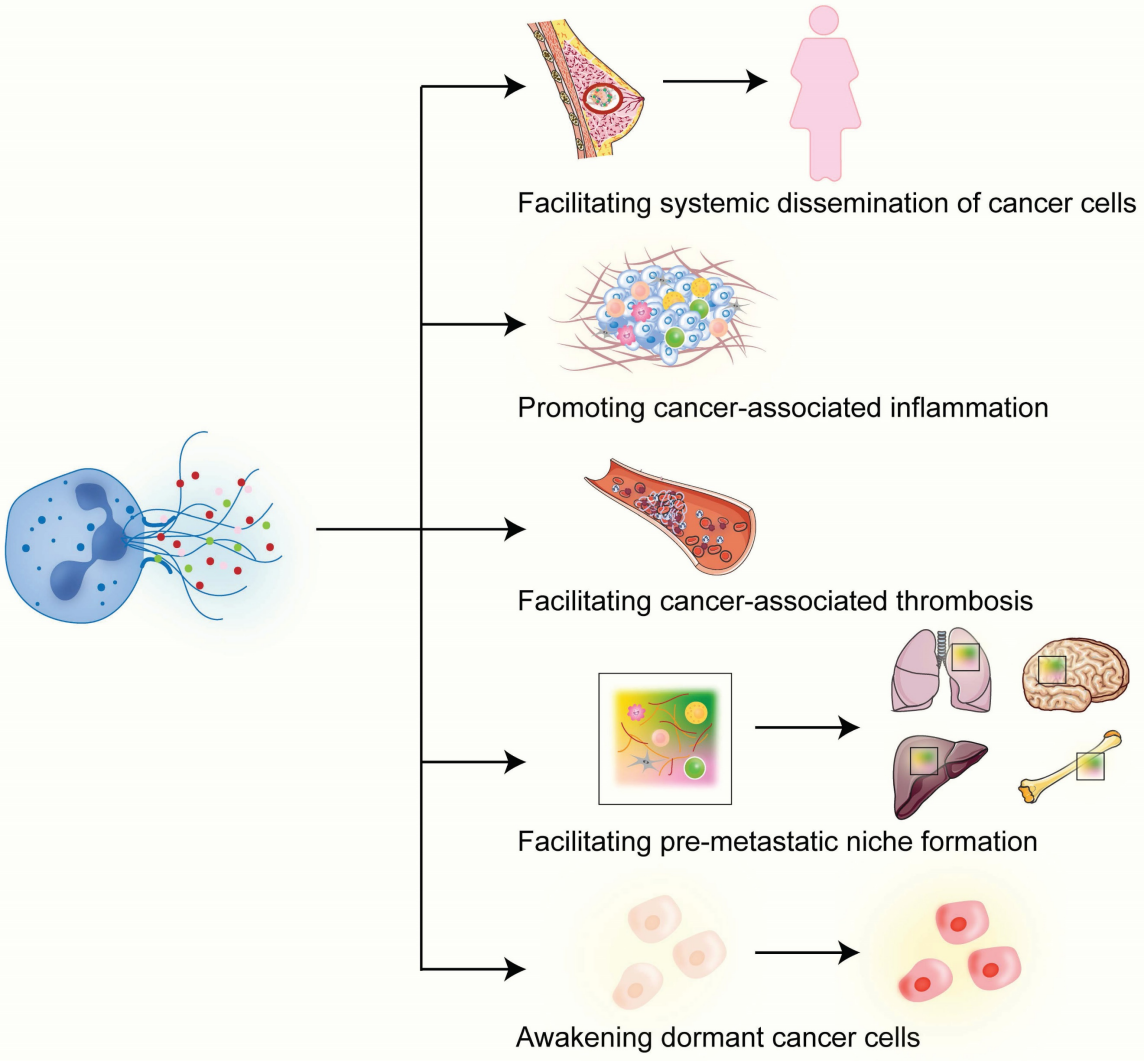
** Roles of NETs in breast cancer metastasis.** In breast cancer, NETs play different functions in various aspects of cancer metastasis. See the main text for detailed description of each of the functions played by NETs in breast cancer metastasis.

Metastasis is a multi-step process in which cancer cells spread from one site to another in the body, often leading to incurability of the disease and adverse outcomes. Levels of circulating NETs are higher in patients with metastatic disease compared with those with localized breast cancer 33. Studies show that NETs play important roles in each step of cancer metastasis. The first connection between NETs and cancer metastasis came from animal studies showing that NETs promote cancer liver metastasis 34, 35. NETs cause alterations in the basement membrane and the ECM and, thus, can enhance the invasive capacity of breast cancer cells 36. In this section, we will delve into the functions and effects of NETs in the various aspects of breast cancer metastasis (**Figure 2**).

### 3.1 Facilitating systemic dissemination of cancer cells

Tumor cells possess the ability to spread to different parts of the body through the bloodstream or the lymphatic system, maintaining their vitality during this journey. They can survive in the circulation and later extravasate 5. It has been suggested that NETs play a critical role in the hematogenous spread of tumors. NETs can induce morphological changes in endothelial cells, leading to increased vascular permeability, which facilitates cancer cell extravasation and promotes breast-to-lung metastasis 37. Kolaczkowska and colleagues found that the attachment of circulating NETs to blood components increases the efficiency of cancer cell extravasation, allowing cancer cells to cross the endothelial barrier 38. Furthermore, NETs may impact the metastatic process through interactions with circulating tumor cells (CTCs). It has been shown that CTCs may become enveloped by the DNA fibers of NETs, leading to their lodging in the liver, thereby forming micro-metastatic foci in a short period 34. A further study has shown that during this process, NETs interact with integrin β1 on the surface of tumor cells, promoting the formation of metastatic foci and the spread of cancer cells 39. The interaction between CTCs and TANs results in endothelial cell contraction, increased permeability, and malignant cell extravasation 40.

#### 3.1.1 NETs promote cancer dissemination through epithelial-to-mesenchymal transition (EMT)

Epithelial-to-mesenchymal transition (EMT) is a process through which epithelial cells acquire mesenchymal properties, endowing cancer cells with invasive and metastatic potential 41. Martins-Cardoso *et al.* found that NET-treated breast cancer cells have increased levels of N-cadherin and fibronectin together with a decreased level of E-cadherin in addition to the morphological changes typical of EMT 42. Since EMT is associated with the cancer stem cell properties, they further found that in addition to promoting EMT, NETs also induce the acquisition of the CD44^high^/CD24^low^ phenotype in MCF7 breast cancer cells, suggestive of enhanced stem cell properties 43.

#### 3.1.2 NET-DNA receptor CCDC25 promotes tumor cell dissemination

The DNA component of NETs (NET-DNA) can act as a chemotactic factor, attracting cancer cells disseminated from the primary site. Yang and colleagues discovered a transmembrane protein called Coiled-Coil Domain-Containing Protein 25 (CCDC25), which serves as a receptor for NET-DNA on cancer cells. CCDC25 is overexpressed in breast cancer and can sense NET-DNA to promote breast cancer liver metastasis 44. In addition to functioning as a receptor for NET-DNA, CCDC25 also activates the ILK-β-PARVIN pathway, attracting cancer cells and enhancing their motility. Furthermore, knockout of the CCDC25 gene in cells has been shown to eliminate NET-mediated breast cancer metastasis 44.

Cholesterol is generated through the mevalonate (MVA) pathway via a series of enzymatic steps. In this process, 3-hydroxy-3-methylglutaryl-CoA reductase (HMGCR) is the rate-limiting enzyme. Cholesterol is generally considered as a risk factor for breast cancer and cholesterol biosynthesis is related to stem cell characteristics in breast cancer 45, 46. One study found that cholesterol biosynthesis is closely associated with NET formation and breast cancer metastasis 47. Furthermore, the expression of CCDC25 in breast cancer tissues is positively correlated with levels of HMGCR and citrullinated histone H3 (H3cit), and high expression of CCDC25 and HMGCR is associated with adverse prognosis in breast cancer patients. Cholesterol biosynthesis promotes the expression of CCDC25 and the formation of NETs in a lipid raft-dependent manner 47. Following treatment with cholesterol biosynthesis inhibitors or DNase I, a significant reduction in CCDC25 expression was observed 47. Additionally, the tumor suppressor gene apoptosis-stimulating of p53 protein 2 (ASPP2) interferes with cholesterol biosynthesis and indirectly affects the expression of CCDC25 and NET formation, weakening the stem cell characteristics, EMT, and invasive capabilities of breast cancer cells. Downregulation of ASPP2 has been shown to promote the transformation of epithelial cells into mesenchymal cells and enhance the invasive abilities of breast cancer cells 48.

#### 3.1.3 Cathepsin C promotes breast cancer lung metastasis through NET formation

Xiao and colleagues found that elevated expression of cathepsin C (also known as dipeptidyl peptidase 1, DDP1), a lysosomal cysteine protease essential for the catalytic activation of several serine proteases, is associated with lung metastatic capabilities in multiple breast cancer cell lines 49, 50. Clinically, high cathepsin C expression is correlated with lung metastasis and decreased overall survival in breast cancer patients 51, 52. The expression and secretion of cathepsin C are related to NET formation and lung metastasis in human breast tumors. Cathepsin C activates the serine protease PR3 on the surface of neutrophils, leading to the processing of IL-1β and the activation of nuclear factor NF-κB in neutrophils. These changes result in the upregulation of IL-6 and CCL3, promoting neutrophil recruitment and triggering an inflammatory cascade and NET formation 51. Simultaneously, cancer cells support the growth of lung metastases through the NET-mediated degradation of TSP-1. Importantly, the second-generation inhibitor of cathepsin C, AZD7986, can effectively disrupt NETs and prevent lung metastasis of breast cancer 51, 53.

#### 3.1.4 NETs play a crucial role in angiogenesis

In addition to the above-mentioned roles played by NETs in breast cancer cells, NETs also play a role in angiogenesis 54. Under stimulation by angiopoietin 1/2, the number of NETs produced by neutrophils is approximately 2.5 times higher than in their natural state, further promoting angiogenesis 55. NETs can damage vascular endothelial cells, activate inflammatory responses, and promote angiogenesis. By evaluating the activity of tumor-related biological pathways, it has been found that NET formation is synchronized with angiogenesis 56, 57. NETs also enhance endothelial cell proliferation and further contribute to the formation of tubular structures 58. It has been demonstrated that histones within NETs promote endothelial cell proliferation and new blood vessel formation. The application of histone-binding agents prevents histone-induced angiogenesis 59.

### 3.2 Promoting cancer-associated inflammation

While neutrophils release NETs to capture pathogens, persistent infections, and inflammatory environments can lead to excessive NET production, thereby promoting the development of various inflammatory diseases. This is primarily mediated through the activation of inflammasomes 19, 60. Similarly, studies have found interactions between inflammasomes and NETs in cancer. It has been demonstrated that NET-associated serine proteases, such as NE, can act as alternative enzymes for processing IL-1β and IL-18 associated with inflammasomes. This subsequently leads to the inactivation of progranulin (PGRN) and the activation of MMP9 in cancer 61, 62. Lu *et al.* also pointed out the relevance of NETs to the feedforward loop of inflammation, which includes the recruitment of inflammatory cells, activation of signaling pathways, and the production of inflammatory factors 63. Inflammation within the tumor microenvironment is crucial for breast cancer and its metastatic development 64-67.

In the liver metastasis of breast cancer, neutrophils release NETs through interactions with platelets and the NF-κB signaling pathway 11, 68, 69. NETs serve as chemoattractants to draw CTCs, thereby promoting liver metastasis. Furthermore, the release of NETs into the circulation can cause endothelial cell damage and platelet activation, stimulating other neutrophils to release NETs 70, 71. Therefore, the occurrence of the inflammatory feedback loop in the liver, driven by NETs, promotes breast cancer liver metastasis. Lu and colleagues utilized a doxorubicin-loaded liposome-encapsulated low-molecular-weight heparin-astaxanthin nanoparticle (LA/DOX NP) to inhibit NET formation, thereby blocking the tumor's inflammatory and immune-suppressive microenvironment and, as a result, inhibiting breast cancer liver metastasis 63. These interventions in the inflammatory feedback loop hold promise as strategies to restrain breast cancer metastasis.

### 3.3 Facilitating cancer-associated thrombosis

Cancer is often associated with a hypercoagulable state and increased thrombosis is one of the most common comorbidities associated with cancer. Indeed, cancer-associated thrombosis (CAT) is the second leading cause of death for cancer patients, second only to the cancer itself 72. It has been shown that chromatin released into the bloodstream through NETs can promote coagulation and thrombus formation 9, 73. Thrombosis leads to poor prognosis in cancer patients and cancer-induced fatalities 9. Mounting evidence suggests that neutrophils and NETs play a significant role in thrombus formation, particularly in CAT, as neutrophils are often reshaped with an increased count, making them more prone to generating NETs in cancer 9, 73-76. It is estimated that cancer patients face a 4-to-7 times higher risk of developing venous thrombosis compared with non-cancer patients 77, 78. Cancer patients who experience venous thrombosis typically have a poorer prognosis 79, 80. Cancer therapies such as surgery, chemotherapy, and angiogenesis inhibitors can further exacerbate CAT 81.

There is significant correlation between elevated NET markers and the hypercoagulable state 5, 59. It has become well-acknowledged that cancer patients exhibit higher levels of NET biomarkers compared with non-cancer patients 82 and cancer-associated NETs promote platelet capturing and increase tissue factor (TF) activity, thereby leading to CAT 74-76, 83. Contrarily, activated platelets can also stimulate the formation of NETs, establishing a positive feedback loop between NETs and CAT 84.

In this context, the inhibition of NETs by drugs or gene manipulation (PAD^-/-^ mice) can reduce thrombosis formation in mice 85-87. Using recombinant human DNase I (rhDNase I) to degrade NETs can prevent thrombosis formation in a mouse model of tumorigenesis. It should be noted that while short-term treatment prevented venous thrombosis formation, long-term treatment reduced survival rates. These results suggest the potential therapeutic efficacy of rhDNase I in treating CAT, but its long-term use needs to be carefully assessed 88. Another study provided initial evidence that low-molecular-weight heparin blocks the adhesion of activated platelets to neutrophils via the P-selectin pathway, thereby inhibiting the generation of NETs 63. Gomes and colleagues also demonstrated that in NET-dependent breast cancer models, inhibiting the production and secretion of IL-1β related to inflammasomes can alleviate CAT 85. These findings suggest that targeting NETs may be a potential and promising approach to reduce thrombosis, limiting tumor progression and metastasis.

### 3.4 Facilitating pre-metastatic niche formation

The pre-metastatic niche (PMN) in cancer typically forms after tumor cells leave the primary site and enter the circulatory system but have not yet reached the metastatic site. This suggests that the PMN is a microenvironment with characteristics of inflammation, immune suppression, and vascular leakage that favor the initiation of metastasis before tumor cells arrive at specific distant organs 89-92. NETs are believed to play a crucial role in the PMN, helping to prepare the "soil" for the "seeds" of metastasis, sequestering CTCs, and promoting metastasis 34, 93. Within the PMN, fibronectin, along with glycoproteins and proteoglycans like tenascin C, osteopontin, and versican, predominantly contributes to the alterations within the ECM 94. NETs play a role in this process, influencing the electrostatic charge and conformation of fibronectin and collagen during citrullination. This effect is mediated by the PAD4 enzyme derived from NETs during the formation of the PMN 10, 95. Furthermore, NETs equipped with proteases are highly correlated with the growth and invasiveness of aggressive tumors. However, this high metastatic potential can be eliminated by DNase I treatment 5, 68, 96.

Recently, there have been studies highlighting the role of NETs in promoting the formation of PMN in breast cancer. Zhou *et al.* found that the loss of the β3 adrenergic receptor (ADRB3) gene affects the PMN of malignant tumor cells, while ADRB3-induced NETs can persist for an extended period and contribute to the generation of PMN, thereby protecting disseminated tumor cells (DTC) from rejection by cytotoxic T lymphocytes 97. In the pre-metastatic stage, the rate-limiting enzyme 2-hydroxyacid oxidase 1 (HAO1) involved in oxalate synthesis is upregulated in the lung alveolar epithelial cells, leading to the accumulation of oxalate in lung tissues. This accumulation activates NOX, inducing neutrophils to produce NETs, thereby promoting the formation of the PMN 98. Furthermore, it has also been shown that resident mesenchymal stem cells (MSCs) play a crucial role in the formation of the PMN in the lungs when breast cancer cells migrate to this organ. MSCs have the ability to recruit neutrophils to the lungs and, with their assistance, generate complement C3 to transform these neutrophils into NETs. A further study has revealed that the upregulation of C3 in the lung's PMN microenvironment during tumor development is driven by Th2 cytokines via the STAT6 signaling pathway. Therefore, there is potential to reduce the likelihood of breast cancer metastasizing to the lungs by intervening in the Th2-STAT6-C3-NET cascade 99.

Another study related to breast cancer metastasis focuses on a subset of neutrophils known as tumor-associated aged neutrophils (Naged, CXCR4^+^ CD62L^low^), which play a critical role in the formation of the pre-metastatic microenvironment. The study used flow cytometry and immunohistochemistry in 206 patients and multiple mouse models to investigate the distribution of Naged. Naged promotes the generation of NETs by activating the histone deacetylase SIRT1 through the tumor-secreted nicotinamide phosphoribosyltransferase (NAMPT), thereby driving tumor metastasis. By intervening in aged neutrophils and their formation pathways, it is possible to effectively reduce breast cancer lung metastasis 100. More recently, using a mouse model of 4T1 breast cancer metastasis, Pan *et al.* demonstrated that chronic stress induces a pre-metastatic niche in the lungs to capture and arrest metastasizing breast cancer cells through the production of acetylcholine (Ach) by pulmonary epithelial cells. Called the CXCL2-Ach-NETosis pathway, this pathway mediates the formation of the lung pre-metastatic niche involves the chemokine CXCL2 that recruits neutrophils into the lungs followed by the production of Ach from pulmonary epithelial cells that enhances NETosis 101. These findings highlight the significant role of NETs in breast cancer metastasis and provide a new direction for the development of targeted therapeutic strategies for this process in the future.

### 3.5 Awakening dormant cancer cells

Cancer cells that have spread from the primary tumor site to other tissues frequently exist in a dormant state and remain relatively silent in those tissues for extended periods. This phenomenon of tumor cell dormancy is common in most solid tumors including breast cancer 102. These dormant cancer cells are often undetectable in clinical settings until they are awakened. Once awakened, these cancer cells can form metastasis and lead to tumor recurrence. It has been demonstrated that NETs have the potential to awaken dormant breast cancer cells, contributing to tumor recurrence and metastatic spread 103. For example, long-term exposure to tobacco smoke or intranasal injection of lipopolysaccharide (LPS), leading to sustained inflammatory conditions, can result in the reinitiation of proliferation and the transformation of dormant breast cancer cells into invasive lung metastases. Even after remaining dormant in the lungs for a month, tumor cells can be awakened by LPS 103.

NETs induce the awakening of dormant cancer cells predominantly via remodeling the extracellular matrix (ECM). It has been demonstrated that proteases within NETs, such as NE and matrix metalloproteinase 9 (MMP9), can cleave and reshape laminin, exposing a binding site that acts as an activation site for integrin α3β1, leading to the activation of FAK/ERK/MLCK/YAP signaling in cancer cells and their reawakening, promoting proliferation. In in vitro experiments, laminins-111, -211, -411, and -511 were identified as critical ECM proteins required for NET-induced awakening of dormant breast cancer cells 103, 104. In addition, thrombospondin-1 (TSP-1), a large glycoprotein present in the basement membrane surrounding mature blood vessels, has also been shown to regulate cancer cell dormancy and metastasis 104-106. Degradation of TSP-1 by NE and MMP9 within NETs 103 helps eliminate the inhibitory effect of TSP-1 on the reawakening of cancer cells, thus assisting in the reawakening of cancer cells. Therefore, both the remodeling of laminins and the degradation of TSP-1 are essential for awakening dormant cancer cells 5, 103.

The cells in proximity to the remodeled laminin typically exhibit a proliferative state, while those near intact laminin remain in a dormant state. The use of specific antibodies to inhibit the new laminin epitopes can impede the reawakening of cancer cells both in vitro and in vivo 103. Additionally, inhibiting the formation of NETs, such as through the use of PAD4 inhibitors or DNase therapy, can prevent the activation and formation of dormant cancer cells. Moreover, in vitro experiments have demonstrated that blocking NE and MMP9 can prevent cancer cells from re-entering the cell cycle and inhibit the progression of LPS-induced cancer in vivo 103. The reawakening of dormant cancer cells is associated with the contribution of NETs to creating a favorable local microenvironment (“soil”) for the colonization of breast tumor cells (“seeds”) 107.

## 4. Translational Implications of NETs in Breast Cancer

### 4.1 NET-related gene signatures as biomarkers for prognosis prediction

Given the important roles of NETs in breast cancer development and progression particularly in the metastatic process, predicting therapeutic responses and patient outcomes using an NET-related gene signature might aid in identification of patients at risk and selection of effective therapies. Zhao *et al.* have explored this 108. Through analyzing the bulk and single-cell RNA sequencing data from public databases, Huang *et al.* constructed a risk index with the NET-related pivotal genes to stratify triple-negative breast cancer patients into high- and low-risk groups. They found that patients of the low-risk group are enriched in Wnt signaling and were more sensitive to Wnt signaling pathway inhibitors. Furthermore, they found that BT549 cells that have a low risk index exhibit higher sensitivity to XVA-939 (a typical Wnt signaling inhibitor) 109. These findings open up new avenue to patient stratification and personalized therapy based on the NET-related gene signature.

### 4.2 NET components as therapeutic targets

**Figure 3:**
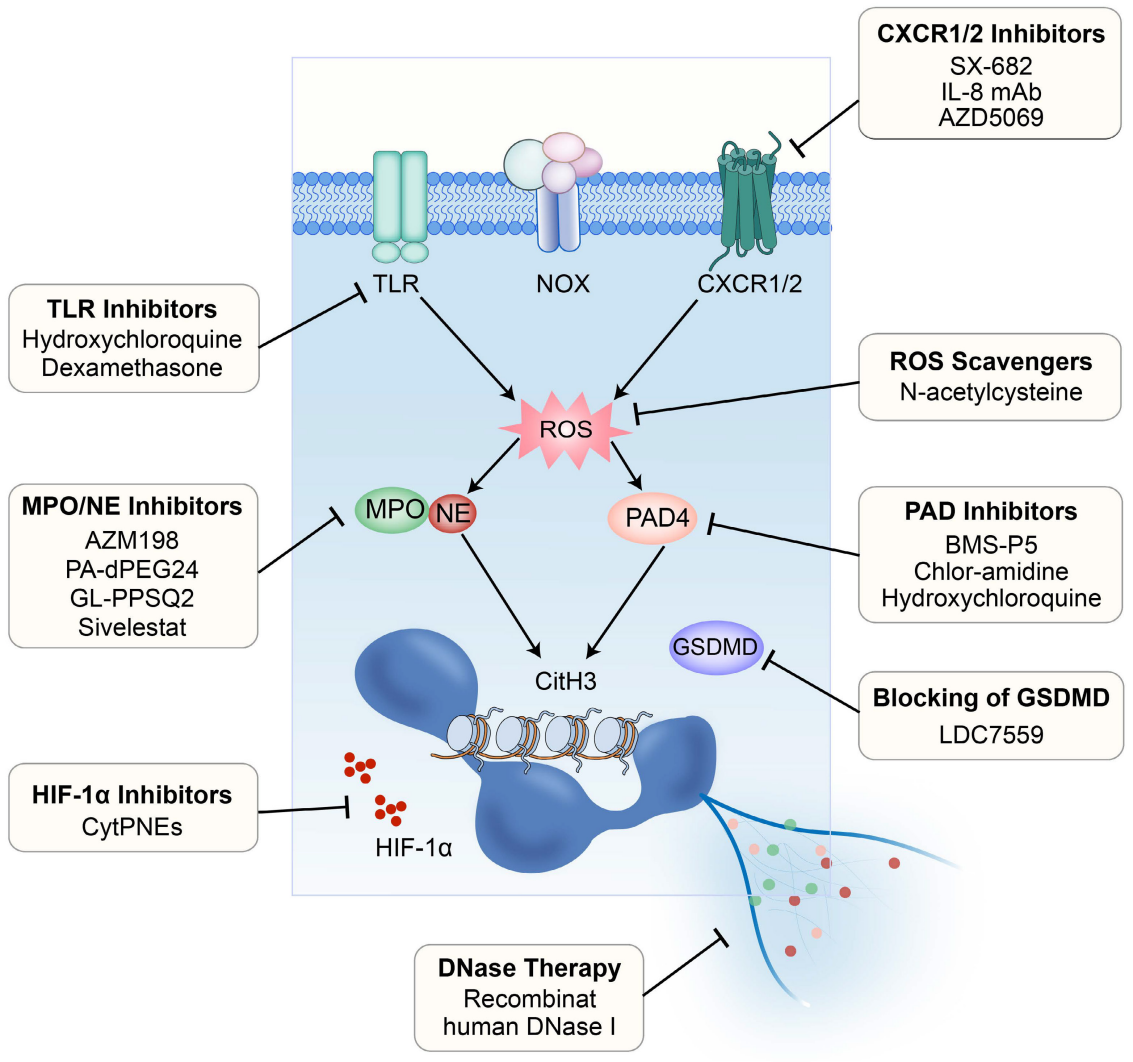
** Strategies for targeting the NETs.** Currently evaluated or potentially available strategies for targeting the NETs to intervene breast cancer metastasis include: 1) inhibition of TLRs; 2) inhibition of CXCR1/2; 3) scavenging of ROS; 4) inhibition of PAD4; 5) blocking of GSDMD; 6) DNase therapy; 7) inhibition of HIF-1α; and 8) inhibition of MPO/NE. TLR, Toll-like receptor; CXCR, CXC chemokine receptor; ROS, reactive oxygen species; PAD4, peptidylarginine deiminase 4; GSDMD, Gasdermin D.

It has been demonstrated that NET formation induced by chemotherapy reduces therapy response in mouse models of breast cancer lung metastasis 110. Therefore, targeting NETs to alleviate therapy resistance through various approaches has become an emerging field of great interest and is expected to be a promising strategy in breast cancer therapy (**Figure 3**).

#### 4.2.1 Targeting the DNA component or its membrane receptor

DNase has the capability to disrupt the structure of the NET by digesting its DNA component, making NETs a potential target for DNase therapy 111. Based on this, DNase has been tested in experimental cancer models. For example, DNase treatment has been shown to disrupt CTC-NET interactions 39 and alleviate the disease burden 95 in experimental models of breast cancer. Park and colleagues further found that using nanoparticles encapsulating DNase I can inhibit the formation of NETs or degrade NETs, significantly reducing lung metastasis in a mouse model of breast cancer 68. It should be noted that excessive inhibition of NETs may weaken the natural defense mechanisms of the immune system and increase the risk of bacterial infections 112. Therefore, Chen and his team have designed a nanoplatform that can release DNase I via photoactivation in a controllable way. This can aid in NET degradation while retaining the interaction between tumor cells and other immune cells. Moreover, the application of this nanoplatform in the liver can prevent the capture of CTCs by NETs, thereby inhibiting metastasis 113.

Another potential intervention strategy may be based on targeting the transmembrane NET-DNA receptor CCDC25, thus reducing cancer invasiveness 44. However, it is important to note that CCDC25 has low tissue specificity, and systemic inhibition of this receptor may have potential side effects, necessitating careful preclinical research. A potential strategy using oncolytic bacterium-mediated delivery of shCCDC25 in vivo may help reduce side effects while sustaining long-term anti-metastasis efficacy, which has shown promising results in a mouse orthotopic model of breast cancer lung metastasis 114.

#### 4.2.2 Targeting the chemokine-receptor interaction

Neutralization of IL-8, a prominent factor triggering the formation of NETs, through monoclonal antibodies or inhibition of its receptors CXCR1/2 using pharmacological inhibitors such as SX-682, has been demonstrated to suppress the release of inflammatory mediators and the formation of NETs 115-117. The use of AZD5069, a small molecule antagonist of CXCR2, has been shown to inhibit the infiltration of neutrophils into brain metastatic foci of breast cancer and may reduce the potential of neutrophils to promote the generation of tumor-associated NETs 118.

#### 4.2.3 Targeting the hypoxic microenvironment

It is well known that tumor development and metastasis are associated with hypoxia, with a concomitant increase in expression and activity of hypoxia-inducible factor-1α (HIF-1α) 119. Simultaneously, ROS generated during NET formation can inhibit the degradation of HIF-1α in tumor cells, leading to HIF-1α accumulation 6, 120. Indeed, accumulation of HIF-1α has been observed in the CTCs of breast cancer patients 121. Researchers have proposed a new strategy to deliver BAY 87-2243, an HIF-1α inhibitor, to cells through the chemotactic action of neutrophils on CTC-NET clusters. By incorporation into a nanoparticle known as cyto-pharmaceuticals (CytPNEs), BAY 87-2243 can be accurately delivered to circulating 4T1 tumor cells, thus suppressing the formation of metastatic foci and extending the survival of mice bearing breast cancer lung metastasis 122.

#### 4.2.4 Targeting the pyroptosis-related pathway

Targeting the pore-forming protein GSDMD, which promotes translocation of NE to the nucleus during suicidal NETosis, has been examined. Through chemical screening, LDC7559 was discovered to specifically bind to GSDMD, reducing the activation of pathological inflammasomes and NETosis 26. Targeting receptors such as the G-CSF receptor (G-CSFR) on tumor cells can also affect the formation of NETs. Wang *et al.* found that anti-G-CSFR monoclonal antibodies possess the ability to inhibit NET release without compromising immune responses against pathogens 123.

## 5. Concluding Remarks and Perspectives

An increasing body of evidence suggests that NETs play a crucial role in breast cancer development, particularly in metastasis. NETs not only facilitate the migration and invasion of breast tumor cells but also promote their survival and growth in the bloodstream as well as in the microenvironment. Understanding the role of NETs in breast cancer development is of great clinical importance. Various treatment strategies targeting NETs are being actively explored, aimed at enhancing immune cell responses and effectively inhibiting the growth and metastasis of breast cancer. However, further research is needed to gain a deeper understanding of the specific mechanisms of NETs in breast cancer and to evaluate the effectiveness and potential side effects of these intervention strategies.

NETs should be regarded as a double-edged sword in the development of cancer of cancer. Although the majority of current studies point to a pro-metastatic role of NETs in breast cancer, we should bear in mind that the NET, a web-like structure as implied by its name, can also trap cancer cells and restrain their spread to distant organs. Therefore, we should be cautious in designing anti-NET therapies against cancer and in explaining the results from these investigations. In addition, we need to bear in mind that most of the NET components are the factors important for normal functioning of neutrophils. Therefore, targeting the NET components may also lead to neutrophil suppression and cause side effects such as neutropenia and increased risk of life-threatening infections. Nevertheless, targeting the NETs provides a promising therapeutic strategy with the potential of restraining the metastatic spread of breast cancer. Further understanding the pathophysiology behind NETs and their obligations in breast cancer metastasis will be instrumental in designing NET-targeting approaches for effective breast cancer therapy.
